# Walking Speed Classification from Marker-Free Video Images in Two-Dimension Using Optimum Data and a Deep Learning Method

**DOI:** 10.3390/bioengineering9110715

**Published:** 2022-11-19

**Authors:** Tasriva Sikandar, Sam Matiur Rahman, Dilshad Islam, Md. Asraf Ali, Md. Abdullah Al Mamun, Mohammad Fazle Rabbi, Kamarul H. Ghazali, Omar Altwijri, Mohammed Almijalli, Nizam U. Ahamed

**Affiliations:** 1 Faculty of Electrical and Electronics Engineering, University of Malaysia Pahang, Pekan 26600, Malaysia; 2 Department of Software Engineering, Daffodil International University (DIU), Dhaka 1341, Bangladesh; 3 Department of Physical and Mathematical Sciences, Chattogram Veterinary and Animal Sciences University (CVASU), Chattogram 4225, Bangladesh; 4 Department of Computer Science, American International University-Bangladesh (AIUB), Dhaka 1229, Bangladesh; 5 Electronics Division, Atomic Energy Centre, Dhaka 1000, Bangladesh; 6 School of Health Sciences and Social Work, Griffith University, Gold Coast, QLD 4222, Australia; 7 Biomedical Technology Department, College of Applied Medical Sciences, King Saud University, Riyadh 11451, Saudi Arabia; 8 Department of Radiation Oncology, School of Medicine, University of Pittsburgh, Pittsburgh, PA 15232, USA

**Keywords:** two-dimensional (2D) image, marker-free video, walking speed, walking speed classification, bi-LSTM, deep learning, redundant feature, ratio-based body measurement, optimal feature

## Abstract

Walking speed is considered a reliable assessment tool for any movement-related functional activities of an individual (i.e., patients and healthy controls) by caregivers and clinicians. Traditional video surveillance gait monitoring in clinics and aged care homes may employ modern artificial intelligence techniques to utilize walking speed as a screening indicator of various physical outcomes or accidents in individuals. Specifically, ratio-based body measurements of walking individuals are extracted from marker-free and two-dimensional video images to create a walk pattern suitable for walking speed classification using deep learning based artificial intelligence techniques. However, the development of successful and highly predictive deep learning architecture depends on the optimal use of extracted data because redundant data may overburden the deep learning architecture and hinder the classification performance. The aim of this study was to investigate the optimal combination of ratio-based body measurements needed for presenting potential information to define and predict a walk pattern in terms of speed with high classification accuracy using a deep learning-based walking speed classification model. To this end, the performance of different combinations of five ratio-based body measurements was evaluated through a correlation analysis and a deep learning-based walking speed classification test. The results show that a combination of three ratio-based body measurements can potentially define and predict a walk pattern in terms of speed with classification accuracies greater than 92% using a bidirectional long short-term memory deep learning method.

## 1. Introduction

Human gait factors of both healthy individuals and patients, such as the stride length, cadence, stance, swing periods, and hip, knee ankle and pelvic tilt joint kinematics, exhibit significant alterations in response to changes in the walking speed [1,2]. For example, healthy individuals exhibit decreases and increases in the amplitudes of cadence, step and stride lengths, stance and swing periods at slower and faster speeds, respectively [3,4]. In addition, changes in walking circumstances do not appear to alter the walking speed of healthy individuals but may have an impact on the walking speed of an individual with a physical impairment who is walking at the same speed. For instance, patients with neurological disorders such as Alzheimer’s disease and neuromuscular problems, including post-stroke and cerebral palsy, exhibit a slower walking speed than healthy controls [5,6,7]. Additionally, in individuals older than 60 years, a slower walking speed is predictive of increased morbidity and mortality [8]. For this reason, walking speed has long been used by clinicians as a straightforward but efficient gait assessment tool for determining demographic traits (such as gender and age) and physical functions including spatiotemporal parameters as well as kinematic and kinetic patterns [5,6,9,10,11]. Most importantly, by combining cutting-edge artificial intelligence techniques (such as deep learning) and conventional video (i.e., two-dimensional [2D] videos or image sequences) surveillance, the walking speed can be used as an independent screening tool for several physical consequences or accidents (e.g., fall-related fear) among healthy individuals and patients with conditions such as Parkinson’s disease and osteoarthritis during day-to-day gait monitoring in healthcare centres and old-age homes. Specifically, body measurement data of walking individuals (e.g., healthy or patients) extracted from 2D marker-free video image sequences can be considered sequential gait data [12,13] for the creation of walk pattern suitable for walking speed classification using artificial intelligence techniques, and the method may be applied in healthcare settings and elderly care facilities [13].

Numerous studies have researched walking gait using body measurements from 2D video or image sequence setups with a focus on speed-related factors and without the use of artificial intelligence approaches [14,15]. The extracted body measurement data from these studies include unilateral hip, knee, ankle and pelvic tilt joint kinematics [14] and body measurement data (e.g., lower-body width) of individuals [15]. However, the clothing worn (i.e., socks and undergarments) by the walking individuals has been employed as segmental markers to monitor foot and pelvic parameters in the image, which results in a significant dependence of the derived body measurement data on the clothing [14]. In addition, the body measurement data from walking individuals, such as height, width, and area, in an image exhibits inconsistent alterations based on the individual’s distance from the camera in various circumstances (e.g., indoor and outdoor settings) [12,15,16]. One strategy to resolve this constraint could be scaling or resizing the video image sequences in order to equalise the walking individual’s body measurements in each image, but doing so may result in visual distortion and reduced quality due to compression and stretching [16]. Another approach for overcoming this limitation could be utilizing the walking individual-to-camera distance independent body measurement data to establish steady walking speed patterns [12]. A study conducted by Zeng and Wang presented body measurement data based on a ratio (i.e., body height-width ratio data) that is steady regardless of the closeness of the individual to the camera while walking [12]. In addition, the study conducted by Zeng and Wang utilized artificial intelligence techniques for classifying walk patterns in terms of speed and established a walking pattern that could be used for classification through the use of inconsistent body measurements (e.g., body area, mid-body and lower-body width) data along with ratio-based (i.e., body height-width ratio) data [12]. Our previous published study [13] provided the first suggestion of five ratio-based body measurements, namely, (i) the ratio of the full-body height to the full-body width (HW1), (ii) the ratio of the full-body height to the mid-body width (HW2), (iii) the ratio of the full-body height to the lower-body width (HW3), (iv) the ratio of the apparent body area to the full-body area (A1), and (v) the ratio of the area between two legs to the full-body area (A2) for the definition and prediction of walk speed patterns. Our previous study [13] then proved the reliability of these five ratio-based body measurements to define and classify an individual’s walking patterns in terms of speed in indoor (treadmill trial) environments using a bidirectional long short-term memory (biLSTM) deep learning-based model with a mean ± standard deviation (SD) classification accuracy of 88.05(±8.85)% and a median accuracy of 89.58%. However, the development of a successful and highly predictive deep learning architecture for walking speed classification depends on the dimension of the data extracted from 2D marker-free video images [17]. Although the use of high-dimensional input features (i.e., several ratio-based body measurements) is thought to create a strong walk pattern, the use of redundant data may overburden the deep learning architecture and hinder the classification performance [18]. Therefore, the use of fewer but useful ratio-based body measurements data from 2D marker-free video images is necessary to build a successful deep learning-based model. Therefore, the current study aimed to construct walk patterns with fewer but useful ratio-based body measurements for the successful development of a deep learning architecture that would classify walking speed with the highest classification accuracy.

One of the commonly used methods for selecting the most beneficial and ideal input features (such as ratio-based body measurements) is assessing the correlations between the features and selecting those with the lowest correlation strengths because only one of two highly correlated input features is needed for a model, while the second feature does not provide any new information for target prediction [19,20]. In other words, the selection of input features with low correlations among them will provide valuable information to a model to improve its predictive ability [20]. Other commonly used methods for optimal input feature selection is fitting and assessing a deep learning-based model with several potential subsets or combinations of input features and selecting the feature subset or combination that yields the best performance [20,21]. The utilization of both methods is crucial for the development of a successful and highly predictive deep learning architecture because an analysis of the correlations among input features will yield theoretical knowledge of the quality (e.g., strong or weak) of the combination of input features, and the practical application of a deep learning-based model using different possible subsets or combinations of input features will identify the feature subset or combination that yields the best performance [19,20,21,22].

The objective of this study was to identify the optimal combination of ratio-based body measurements needed for presenting potential information that can define and predict a walk pattern in terms of speed with high classification accuracy using a deep learning-based walking speed classification model. To this end, the study analysed the correlations among five ratio-based body measurements to comprehend the relationships among ratio-based body measurements in slow, normal and fast walking speed conditions. This study also evaluated the performance (in terms of the mean ± SD classification accuracy and mean ± SD training time) of a biLSTM deep learning-based walking speed classification model using the walking speed patterns created by all possible combinations of one, two, three and four ratio-based body measurements among five ratio-based body measurements (HW1, HW2, HW3, A1, and A2). The walk pattern created by the combination of fewest ratio-based body measurements (i.e., less than five ratio-based body measurements) was defined as optimal in the study if it was able to classify the walking speed with a mean ± SD classification accuracy higher than or within 2% less [23,24] of that obtained in our previous study [13], and the ratio-based body measurements in the walk pattern showed low correlations among them. This study hypothesized that walking speed patterns identified from few ratio-based body measurements can be used to classify walking speed using deep learning-based methods with high accuracy if the correlations among the body measurements are low.

## 2. Methods

This study adopted lateral 2D marker-free motion image sequences from a publicly available dataset, the Osaka University-Institute of Scientific and Industrial research (OU-ISIR) dataset ‘A’ [25]. This is a benchmark dataset and has been used in various research areas since it was publicly published in 2012. The dataset has been used in the area human gait research focusing on speed, age, and gender [12,26], movement assessment and gait monitoring [13,27], gait-based biometric and surveillance [28,29].

### 2.1. Participants and Dataset

**Figure 1:**
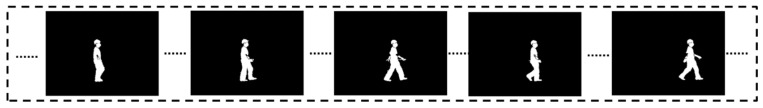
Example of continuous image sequences from OUISIR dataset A for one participant walking at a normal speed.

In this study, the walk speed patterns at three speeds—slow, normal, and fast—were classified using lateral 2D marker-free motion image sequences from 34 participants. The OU-ISIR dataset ‘A’ [25], which is available publicly, provided these image sequences (obtained using an indoor treadmill) (Figure 1). Three walking speed categories were considered: slow (2 to 3 km/h), normal (4 to 5 km/h) and fast (6 to 7 km/h) [30,31,32]. OU-ISIR dataset ‘A’ comprises of 2D image sequences recorded from 34 participants while walking at a range of speed from 2 to 7 km/h on a 550 mm wide and 2000 mm long belt area of treadmill (BIOMILL BM-2200). An increment of 1 km/h speed was maintained consistently. All participants wore standard coloured long sleeve shirt and long pants while walking. The lateral view image sequences of the participants were captured using camera (Point Grey Research Inc. Flea2 models) with 3.5 mm lens focal length, 60 fps frame rate and VGA resolution. The image sequence data were divided into the three above-mentioned categories (i.e., slow, normal, and fast). Additionally, the dataset included both male and female participants with age between 15 to 65 years who had reported no recent fall injuries, neurology or orthopaedic and gait or locomotion related issues. For each participant, 12 image sequences including two image sequences for each speed were processed, that yielded a total of 408 sequences with a minimum length of 240 frames. Three types of walk speed patterns for slow, normal and fast walking were created using quasi-periodic patterns produced from five ratio-based body measurements extracted from the minimum number of image sequences (i.e., 240 frames), which are comparable to the lengths used in previous studies [13].

### 2.2. Feature Extraction

**Figure 2:**
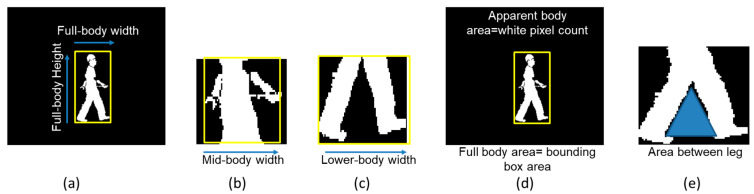
Detail of the terms used in Equations (1)–(5). Extraction of (**a**) full-body height (H) and full body width (W1) (**b**) mid-body width (W2) (**c**) lower-body width (W3) (**d**) full body area and apparent body area, and (**e**) area between two legs.

According to the procedure used in our prior study [13], which is depicted in Figure 2 and exemplified by Equations (1)–(5), data for five ratio-based body measurements (HW1, HW2, HW3, A1 and A2) were extracted from image sequences available for slow walk, normal walk, and fast walking. More specifically, among the five ratio-based body measurements defined in our previous study [13], HW1, HW2 and HW3 were calculated using the rectangular boundary box height and width. Bounding boxes were placed around the whole body, mid body and lower body locations in each image, and HW1, HW2 and HW3 were then calculated using Equations (1)–(3). The terms in the equations are presented in Figure 2a–c. A1 and A2 were measured by evaluating the white pixels in the image, boundary box area and area between two legs in each image and then using Equations (4) and (5). The terms in the equations are presented in Figure 2d,e.

Ratio of the full-body height to the full-body width,

(1) $$\mathrm{HW}1=\frac{\mathrm{Full}-\mathrm{body}\;\mathrm{height}}{\mathrm{Full}-\mathrm{body}\;\mathrm{width}}$$

Ratio of the full-body height to the mid-body width,

(2) $$\mathrm{HW}2=\frac{\mathrm{Full}-\mathrm{body}\;\mathrm{height}}{\mathrm{Mid}-\mathrm{body}\;\mathrm{width}}$$

Ratio of the full-body height to the lower-body width,

(3) $$\mathrm{HW}3=\frac{\mathrm{Full}-\mathrm{body}\;\mathrm{height}}{\mathrm{Lower}-\mathrm{body}\;\mathrm{width}}$$

Ratio of the apparent body area to the full-body area,

(4) $$A1=\frac{\mathrm{Apparent}-\mathrm{bodyarea}}{\mathrm{Full}-\mathrm{bodyarea}}$$

Ratio of the area between two legs to the full-body area,

(5) $$A2=\frac{\mathrm{Area}\;\mathrm{between}\;\mathrm{two}\;\mathrm{legs}}{\mathrm{Full}-\mathrm{bodyarea}}$$

**Figure 3:**
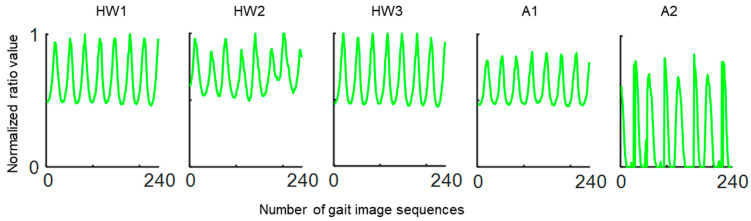
Quasi-periodic signals created by five ratio-based body measurements calculated from image sequences of a single individual moving normally while walking. HW1, ratio of the full-body height to the full-body width; HW2, ratio of the full-body height to the mid-body width; HW3, ratio of the full-body height to the lower-body width; A1, ratio of the apparent body area to the full-body area; and A2, ratio of the area between two legs to the full-body area.

After extracting data for five ratio-based body measurements from marker-free 2D image sequences, our previous research [13] discovered that each of the five ratio-based body measurements varied over time such that they created quasi-periodic patterns (Figure 3), which is an established pattern of human gait cycle motion while walking [33].

### 2.3. Experiment Procedure

In the current study, for each walking speed condition, coefficient of determination (R^2^) were calculated among the data of five ratio-based body measurements to determine the ratio-based body measurements with low correlation. R-Square (R^2^) has been used as a state-of-the-art tool for correlation analysis [34]. The results from the correlation analysis are presented in terms of R^2^ in Section 3. The quasi-periodic patterns were then used to establish three types of walk speed patterns for slow, normal and fast walking. Thirty datasets were created using three types of walk speed patterns. Among these datasets, the walk speed patterns in five, ten, ten and five datasets were established using quasi-periodic patterns from one, two, three and four of the five ratio-based body measurements, respectively. The combinations of ratio-based body measurements in the walk patterns obtained with the above-described datasets were established according to the combination rule in Equation (6), and no combinations were repeated for different orders of ratio-based body measurements. This process of creating a combination of features have been used by the current studies [35,36].

(6) $$C_{n}=\frac{5!}{n\;!(5-n)!},\;n=1,2,\ldots ,4$$

In this equation, *C*(*n*) is the number of combinations generated by the included ratio-based body measurements, 5 is the total number of ratio-based body measurements, n is the number of included ratio-based body measurements in the combination, and (5 − n) is the number of ratio-based body measurements excluded from the combination.

**Figure 4:**
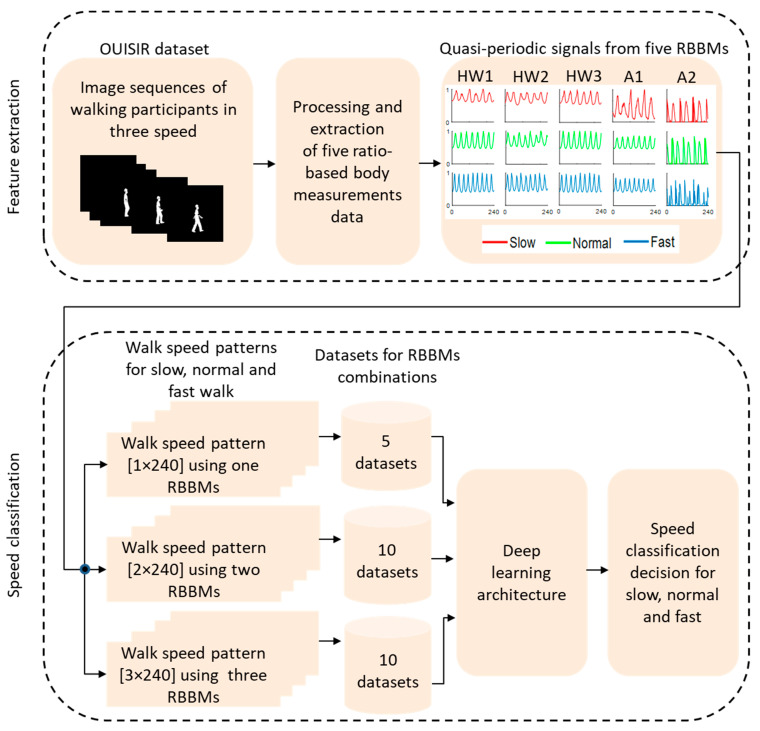
Workflow of the walking speed classification using different combinations of ratio-based body measurements (RBBMs).

Each dataset contained 136 walk speed patterns for each of the three speeds (i.e., slow, normal, and fast). Table 1 provides a description of the walk patterns in all the datasets. After datasets’ construction, a biLSTM-based deep learning architecture along with k-fold (where, k = 17) cross validation [13] was performed using all ratio-based body measurements combinations (Table 1) for walking speed classification. A total of 272 cross validation experiments were performed for each deep learning-based walking speed classification task. According to the prior studies, this simple structure is adequate to produce non-overfitting and highly accurate classification problems of the same types [37,38]. Figure 4 presents workflow of the walking speed classification using different combination of ratio-based body measurements. The results from the walking speed classification are presented in terms of mean ± SD classification accuracies and mean ± SD training time in Section 3 and in Supplementary Material (Tables S1–S5).

## 3. Results

**Figure 5:**
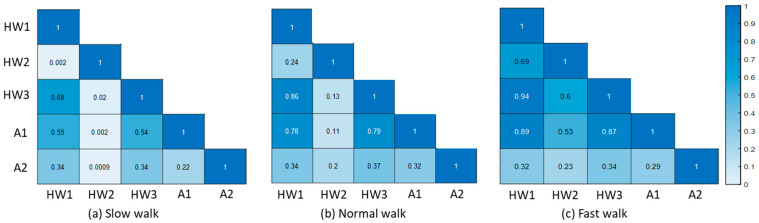
Coefficient of determination (R^2^) among data of five ratio-based body measurements for (**a**) slow (**b**) normal and (**c**) fast walk speeds. HW1, ratio of the full-body height to the full-body width; HW2, ratio of the full-body height to the mid-body width; HW3, ratio of the full-body height to the lower-body width; A1, ratio of the apparent body area to the full-body area; and A2, ratio of the area between the legs to the full-body area. Weak correlation: 0.10–0.39, moderate correlation: 0.40–0.69, strong correlation: 0.70–0.89 and very strong correlation: 0.90–1.00.

Figure 5 presents the results from the correlation analysis (in terms of R^2^) using data of five ratio-based body measurements for slow, normal and fast walk speeds. According to the interpretations (i.e., weak correlation: 0.10–0.39 and moderate correlation: 0.40–0.69, strong correlation: 0.70–0.89, very strong correlation: 0.90–1.00) [39], the R^2^ values between HW1 vs. HW2, HW2 vs. HW3, HW2 vs. A1, HW1 vs. A2, HW2 vs. A2, HW3 vs. A2 and A1 vs. A2 were generally found to be weak for slow and normal walk speeds, whereas for fast walk speeds, weak and moderate R^2^ values were found between HW1 vs. A2, HW2 vs. A2, HW3 vs. A2 and A1 vs. A2 and between HW1 vs. HW2, HW2 vs. HW3, and HW2 vs. A1, respectively. In addition, moderate R^2^ values were found between HW1 vs. HW3, HW1 vs. A1, and HW3 vs. A1 for slow walk speeds, but the corresponding values obtained for normal and fast walk speeds were generally strong.

**Figure 6:**
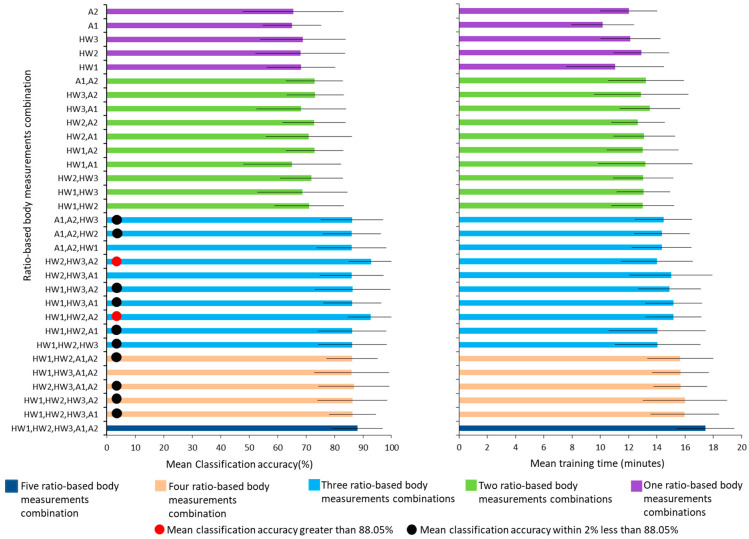
Mean ± SD classification accuracy and mean ± SD training time for biLSTM-based walking speed classification using walk speed patterns based on by one, two, three, four and five ratio-based body measurements. HW1, ratio of the full-body height to the full-body width; HW2, ratio of the full-body height to the mid-body width; HW3, ratio of the full-body height to the lower-body width; A1, ratio of the apparent body area to the full-body area; and A2, ratio of the area between the legs to the full-body area.

Figure 6 presents the results from comparisons of the mean(±SD) classification accuracy and mean(±SD) training time for biLSTM-based walking speed classification using walk speed patterns established using one, two, three four and five ratio-based body measurements. Details of the mean(±SD) classification accuracy and mean(±SD) training time are provided given in the Supplementary Material (Tables S1–S5). Walking speed classification using walk speed patterns established using five ratio-based body measurements achieved a mean(±SD) classification accuracy of 88.05(±8.85)% (Figure 6 and Table S1 (result from our previous study [13])) and the walk speed patterns established using three ratio-based body measurements combinations such as (HW1, HW2, A2) and (HW2, HW3, A2) achieved a mean classification accuracy that was greater than that achieved with walk speed patterns established with five ratio-based body measurements (Figure 6 and Table S3). More specifically, two combinations of three ratio-based body measurements, namely, (HW1, HW2, A2) and (HW2, HW3, A2), achieved mean(±SD) classification accuracies of 92.7(±8.01)% and 92.79(±7.8)%, respectively (Figure 6 and Table S3). In addition, the walk speed patterns established using other combinations of three ratio-based body measurements, namely, (A1, A2, HW3), (A1, A2, HW2), (HW1, HW3, A2), (HW1, HW3, A1), (HW1, HW2, A1) and (HW1, HW2, HW3), and three combinations of four ratio-based body measurements, namely, (HW1, HW2, A1, A2), (HW1, HW2, HW3, A1) and (HW1, HW2, HW3, A2), achieved mean classification accuracies that were very close (i.e., within 2% less) to the mean classification accuracy achieved with the walk speed patterns established with five ratio-based body measurements (Figure 6 and Tables S2 and S3). In contrast, the mean accuracies achieved for walking speed classification using walk speed patterns established with combinations of one and two ratio-based body measurements were less than 70% and 74%, respectively (Figure 6 and Tables S4 and S5). These results clearly show that the walk speed patterns established with combinations of three ratio-based body measurements achieved better performance in terms of the mean(±SD) classification accuracy than the walk speed patterns established with five ratio-based body measurements. Moreover, the mean training time for walking speed classification using walk speed patterns established with combinations of three ratio-based body measurements reduced to approximately 14 to 15 min (Figure 6 and Table S3) compared with the mean training time of 17.43 min for walking speed classification using walk speed patterns established with the combination of five ratio-based body measurements [Figure 6 and Table S1 (result from our previous published study [13])].

## 4. Discussion

The primary objective of this study was to determine the optimal ratio-based body measurement combination needed to present potential information that can define and predict walk patterns in terms of speed with a high classification accuracy. To accomplish the goal, this study adopted two commonly used methods of useful and optimal selection of input features (e.g., ratio-based body measurements). First, this study analysed the correlations among five ratio-based body measurements to comprehend relationships among these body measurements in slow, normal and fast walking speed conditions. Second, the performance (in terms of the mean ± SD classification accuracy and mean ± SD training time) of a biLSTM deep learning-based walking speed classification model was evaluated using walking speed patterns created using all possible combination of one, two, three and four out of five ratio-based body measurements. The combination with the fewest ratio-based body measurements (i.e., less than five ratio-based body measurements) for the establishment of walk patterns was deemed optimal if it yielded a mean ± SD classification accuracy higher than or within 2% less [23,24] of the mean ± SD classification accuracy obtained in our previous study [13], and the ratio-based body measurements used for defining the walk pattern exhibited low correlations among them.

This study utilized data for five ratio-based body measurements for the correlation analysis and biLSTM deep learning-based walking speed classification. Based on the correlation analysis and biLSTM deep learning-based walking speed classification models, this study discovered that combinations of three ratio-based body measurements with minimal correlation among them yielded the highest accuracy in terms of the mean ± SD classification accuracy for walking speed classification using the biLSTM deep learning-based model. More specifically, HW1 exhibits low correlations with HW2 and A2, and thus, the combination of these three ratio-based body measurements achieved classification accuracy of 92.7(±8.01)% (Figure 5 and Figure 6 and Table S3). HW2 has low correlations with HW3 and A2, and the combination of these three ratio-based body measurements achieved a classification accuracy of 92.79(±7.8)% (Figure 5 and Figure 6 and Table S3). Furthermore, the mean ± SD classification accuracies achieved with the combinations of one and two ratio-based body measurements with low correlation among them are markedly lower than the mean ± SD classification accuracy achieved in our previous study [13] (Figure 6 and Tables S4 and S5). Moreover, the other combinations of ratio-based body measurements achieved classification accuracies within 2% of the mean ± SD classification accuracy achieved in our previous study [13], and the body measurements in these combinations generally exhibited moderate to strong correlations between them (Figure 5 and Figure 6 and Tables S1–S3). This finding implies that walking speed patterns identified from few ratio-based body measurements can produce the best performance for deep learning-based classification of walking speed if the correlation between the ratio-based body measurements is low. Additionally, full body image sequences are necessary for more accurate classification, since ratio-based body measurements (i.e., HW1, HW2 and HW3) which resulted in excellent classification accuracy required full-body height.

This study is significant in several contexts. First, video image sequences display apparent body measurements rather than real physiological dimensions of the human body [12,15,16]. It is thus crucial to examine different walking individual-to-camera distance independent body measurements (i.e., ratio-based body measurements) that can be found from video image sequences and to investigate the interactions between ratio-based body measurements in order to identify the optimal body measurements for defining and predicting a walk pattern in terms of speed [12,13]. By performing a correlation analysis and a rigorous deep learning-based assessment, the current study evaluated combinations of three out of five potential ratio-based body measurements. Combinations of these three ratio-based body measurements provided information to estimate walk patterns in terms of speed with classification accuracy greater than 92%, which is better than the results achieved in previous studies 88.57% [12], 88.05% [13]. In addition, the previous study [12] trained the model with a multiclass setting (i.e., all three types of walking speed patterns) and tested the models using a single-class setting (i.e., any one of the three walking speed patterns) while the current study used a multiclass setting as well as multiple runs for the training, validation and testing of the model, which is beneficial for achieving accurate classification accuracy and building a successful model [40,41]. It is difficult to compare our results with the previously published study [14], which used body-worn clothing for body measurement extraction, as the study only proposed extraction methods and did not experiment for classification related tasks. Additionally, the data collection procedure, experimental design, and participants’ demographic characteristics of the previous study [14] are completely different from the current study. Second, earlier studies [17,18], which claim that using high-dimensional input features (such as several ratio-based body measurements) may hinder the performance of a deep learning-based architecture obtained with redundant data, support the results from the current study. In addition, previous studies [17,18], which assert that the highest performance of a deep learning-based architecture could be attained if the best data that provide information, are in agreement with the results from the current study. Furthermore, in future clinicians may utilise this method for routine gait monitoring in healthcare and old-age homes as it can be used to identify the walking speed in an indoor environment with improved classification accuracy [42]. Current patient monitoring systems include implanted devices and wearable sensors that might require invasive procedures and body attachment which are difficult and often unpleasant for patients. Therefore, remote patient monitoring using existing surveillance cameras could be a more viable option to constant observation of patient mobility. In addition, human resources and battery life of traditional sensors are critical for long term patient monitoring. As such, camera-based patient mobility monitoring might be more cost effective while alleviating the burden on resources in clinical settings [43].

Although the current study has a lot of potential for selecting the optimal ratio-based body measurements for creating walk patterns that are useful for accomplishing walking speed classification using a deep learning-based architecture with the highest classification accuracy, the study only evaluated healthy individuals. Experiments that include a gait-impaired population will be considered in the future. Additionally, this study recruited participants with a wide range of ages (15 to 65). However, the walk patterns of the participants might change according to their age [44,45]. Walk speed classification across different aged participants could be another research topic of interest in future. Additionally, this study solely used area-based and height-to-width ratio-based body measurements for the classification of walking speeds. Future studies will involve estimating additional spatiotemporal parameters, such as stride and step length, joint angles, velocity and acceleration, to gain a deeper understanding of the health of individuals and to classify typical and atypical gait patterns. Moreover, only the biLSTM approach was used in this study for the classification task. Future research will utilise more cutting-edge classification algorithms to reach the best classification accuracy.

## 5. Conclusions

In summary, this study found that combinations of three ratio-based body measurements extracted from lateral-view 2D images of marker-free walking individuals can potentially define and predict walk patterns in terms of speed with classification accuracies greater than 92% using a biLSTM. The excellent findings of this study support the optimal application of ratio-based body measurement data that change with variations in the walking speeds, form periodic or quasi-periodic patterns, and, more importantly, can be extracted from marker-free conventional camera images to classify walking speeds with high classification accuracy using the contemporary deep learning method. Additionally, the remarkable results obtained in this study confirm that the use of high-dimensional input features, such as multiple ratio-based body measurements, hinders the performance of deep learning-based architectures if the data are redundant. Furthermore, if the data that yield the best information are employed, the deep learning-based architecture would exhibit peak performance. This walking speed classification method using optimal data is a simple yet effective technique with a lot of potential for use in clinical settings and elderly care facilities.

## Figures and Tables

**Table 1:** Description of the walk patterns in all datasets used in biLSTM-based deep learning architecture.

No. of Datasets	No. of Ratio-Based Body Measurement in Walk Speed Pattern	Combinations of Ratio-Based Body Measurement in Walk Speed Pattern	Walking Speed Pattern Dimension	No. of Walk Speed Patterns/Dataset			
				Slow Speed	Normal Speed	Fast Speed	Total
05	01	HW1	1 × 240	136	136	136	408
		HW2					
		HW3					
		A1					
		A2					
10	02	HW1, HW2	2 × 240	136	136	136	408
		HW1, HW3					
		HW2, HW3					
		HW1, A1					
		HW1, A2					
		HW2, A1					
		HW2, A2					
		HW3, A1					
		HW3, A2					
		A1, A2					
10	03	HW1, HW2, HW3	3 × 240	136	136	136	408
		HW1, HW2, A1					
		HW1, HW2, A2					
		HW1, HW3, A1					
		HW1, HW3, A2					
		HW2, HW3, A1					
		HW2, HW3, A2					
		A1, A2, HW1					
		A1, A2, HW2					
		A1, A2, HW3					
05	04	HW1, HW2, HW3, A1	4 × 240	136	136	136	408
		HW1, HW2, HW3, A2					
		HW2, HW3, A1, A2					
		HW1, HW3, A1, A2					
		HW1, HW2, A1, A2					

## Data Availability

The data generated and/or analyses for the current study are available from the following publicly available databases: Osaka University-Institute of Scientific and Industrial research (OU-ISIR) Dataset ‘A’: (www.am.sanken.osaka-u.ac.jp/BiometricDB/GaitTM.html, access on 23 September 2022).

## Supplementary Materials

The following supporting information can be downloaded at: https://www.mdpi.com/article/10.3390/bioengineering9110715/s1, Table S1: classification accuracies for walking speed classification using walk pattern established with five RBBMs in our previous study, Table S2: classification accuracies for walking speed classification using walk pattern established with four RBBMs, Table S3: classification accuracies for walking speed classification using walk pattern established with three RBBMs, Table S4 classification accuracies for walking speed classification using walk pattern established with two RBBMs, Table S5 classification accuracies for walking speed classification using walk pattern established with one RBBMs. RBBMs refers to ratio-based body measurements.
