# Supplementary material for: Walking Speed Classification from Marker-Free Video Images in Two-Dimension Using Optimum Data and a Deep Learning Method
Source: Bioengineering (Basel). 2022 Nov 19;9(11):715. doi: 10.3390/bioengineering9110715 (PMC9687360; doi:10.3390/bioengineering9110715)
Supplement: Supplementary file 1 [file bioengineering-09-00715-s001.zip › bioengineering-1960248-supplementary.pdf]

Supplementary material

Table S1 classification accuracies for walking speed classification using walk pattern established with five RBBMs in our previous study [13].

RBBMs combination	Mean(±SD) accuracy (%)	Median accuracy (%)	Mean(±SD) training time (mint.)
HW1, HW2, HW3, A1, A2	88.05(±8.85)	89.58	17.43(±2.03)

study [13] refers to our previous study T. Sikandar *et al.*, "Using a Deep Learning Method and Data from Two-Dimensional (2D) Marker-Less Video-Based Images for Walking Speed Classification," *Sensors*, vol. 21, no. 8, p. 2836, 2021.

Table S2 classification accuracies for walking speed classification using walk pattern established with four RBBMs.

RBBMs combination	Mean(±SD) accuracy (%)	Median accuracy (%)	Mean(±SD) training time (mint.)
HW1,HW2,HW3,A1	86.34(±8.22)	88.1	15.97(±2.43)
HW1,HW2,HW3,A2	86.33 (±12.22)	88.21	15.99(±2.97)
HW2,HW3,A1,A2	86.78 (±12.45)	87.55	15.67(±1.89)
HW1,HW3,A1,A2	86.01 (±13.1)	87.28	15.68(±2)
HW1,HW2,A1,A2	86.22 (±9.01)	88.01	15.66 (±2.34)

Table S3 classification accuracies for walking speed classification using walk pattern established with three RBBMs.

RBBMs combination	Mean(±SD) accuracy (%)	Median Accuracy (%)	Avg. training time (mint.)
HW1,HW2, HW3	86.26 (±12.1)	87.78	14.05(±3.03)
HW1, HW2, A1	86.12(±12.01)	87.01	14.03(±3.44)
HW1, HW2, A2	92.7(±8.01)	94.87	14.12(±1.97)
HW1, HW3, A1	86.26(±10.22)	88.1	15.18(±2.01)
HW1, HW3, A2	86.33(±13.22)	88.01	14.89(±2.22)
HW2, HW3, A1	86(±11.22)	87.23	15.01(±2.93)
HW2, HW3, A2	92.79(±7.8)	95	14.01(±2.53)
A1, A2, HW1	86(±12.22)	88.23	14.35(±2.11)
A1, A2, HW2	86.1(±10.22)	89	14.35(±1.97)
A1, A2, HW3	86.12(±10.99)	87.44	14.47(±2.01)

Table S4 classification accuracies for walking speed classification using walk pattern established with two RBBMs.

**RBBMs combination**	**Mean(±SD) accuracy (%)**	**Median accuracy (%)**	**Avg. training time (mint.)**
HW1,HW2	71.12(±12.09)	72.97	13.00(±2.22)
HW1,HW3	68.79 (5.81)	70.1	13.05(±1.89)
HW2,HW3	71.9(11.04)	72.97	13.03(±2.12)
HW1, A1	65.15(17.11)	67.09	13.18(±3.34)
HW1, A2	73.01(10.01)	75.85	13.01(±2.53)
HW2, A1	71(15.07)	73	13.10(±2.17)
HW2, A2	72.89(11)	73.1	12.67(±1.89)
HW3, A1	68.32(15.67)	70.03	13.50(±2.12)
HW3, A2	73.22(9.97)	75.71	12.89(±3.34)
A1, A2	73(10)	75.8	13.23(±2.67)

Table S5 classification accuracies for walking speed classification using walk pattern established with one RBBMs.

**RBBMs combination**	**Mean(±SD) accuracy (%)**	**Median accuracy (%)**	**Avg. training time (mint.)**
HW1	68.26(±12.05)	70.01	11.05(±3.44)
HW2	68.02(±15.78)	69.72	12.92(±1.97)
HW3	68.89(±15.02)	69.85	12.12(±2.11)
A1	65.13(±10.24)	67.55	10.18(±2.22)
A2	65.51(±17.59)	67.92	12.01(±2.01)
